# Cognitive Profile and Its Evolution in a Cohort of Multiple System Atrophy Patients

**DOI:** 10.3389/fneur.2020.537360

**Published:** 2020-11-23

**Authors:** Luisa Sambati, Giovanna Calandra-Buonaura, Giulia Giannini, Ilaria Cani, Federica Provini, Roberto Poda, Federico Oppi, Michelangelo Stanzani Maserati, Pietro Cortelli

**Affiliations:** ^1^IRCCS, Istituto delle Scienze Neurologiche di Bologna, Bologna, Italy; ^2^Dipartimento di Scienze Biomediche e NeuroMotorie (DIBINEM), Università di Bologna, Bologna, Italy

**Keywords:** mild cognitive impairment, multiple system atrophy (MSA), cognition, neuropsychology, dementia

## Abstract

**Introduction:** Cognitive decline is not a characteristic feature of multiple system atrophy (MSA), but recent evidence suggests cognitive impairment as an integral part of the disease. We aim to describe the cognitive profile and its progression in a cohort of patients with MSA.

**Methods:** We retrospectively selected patients referred to our department with a clinical diagnosis of MSA who were evaluated at least once a year during the course of the disease and underwent a comprehensive neuropsychological evaluation.

**Results:** At the first evaluation (T0), 37 out of 60 patients (62%) were cognitively impaired, mainly (76%) in attention and executive functioning. Thirteen patients were impaired in one cognitive domain and 24 in more than one cognitive domain. Six out of the 24 had dementia. Twenty patients underwent a follow-up evaluation (T1) after a mean of 16.6 ± 9.3 months from the first evaluation (T0). Eight out of 20 patients were cognitively normal at both T0 and T1. Seven out of 12 patients presented with stable cognitive impairment at T1, while cognitive decline progressed in five patients. Patients with progression in cognitive decline performed significantly worse at T0 than cognitively stable patients. Education was significantly different between patients with and without cognitive impairment. No other differences in demographic and clinical variables and autonomic or sleep disturbances were found. Patients with dementia were older at disease onset and at T0 and had lower education and disease duration at T0 compared to those in other groups.

**Conclusions:** In patients with MSA, we observed three different cognitive profiles: normal cognition, stable selective attention-executive deficits, and progressive cognitive deficits evolving to dementia. The detection of cognitive impairment in patients with suspected MSA suggests the need for comprehensive and longitudinal neuropsychological evaluation.

## Introduction

Multiple system atrophy (MSA) is a sporadic neurodegenerative disease characterized by autonomic failure associated with a combination of cerebellar and/or parkinsonian signs. It is subclassified into a cerebellar (MSA-C) or parkinsonian (MSA-P) variant, depending on the predominant clinical phenotype (1).

According to the diagnostic criteria for MSA, dementia is a non-supporting feature for diagnosis (1). However, three cross-sectional studies estimated a dementia prevalence of 15% during the course of the disease (2–4).

An evidence-based review of the “Neuropsychology Task Force of the Movement Disorders Society MSA” (MODIMSA) Study Group suggests cognitive impairment (CI) as an integral feature of the disease (5). However, the frequency of CI varies largely among studies (between 33 and 83%).

Furthermore, due to the heterogeneity of the neuropsychological assessment, the profile of CI in MSA has not been well-characterized. Although executive function seems to be the most impaired function, memory and visuospatial deficits may also occur (5).

Similarly, comparative studies of the association between CI and MSA subtypes in MSA-P and MSA-C led to controversial results (5–10). Considering observational studies, Chang et al. reported comparable neuropsychological performance in both MSA motor subtypes while others (5) reported differences in both the number and type of domains impaired.

It has to be considered that only a few studies evaluated the progression of cognitive deficits on small samples and in short follow-up periods, reporting progressive worsening of speed, attention, and executive function (7, 11–14).

Only one study reported differences between MSA-P and MSA-C at 1 year evaluation, with a significant worsening in both groups but with a different cognitive evolution. In detail, worsening in spatial planning and psychomotor speed was observed in patients with MSA-C and a significant worsening in prose memory, spatial planning, and repetition abilities in patients with MSA-P (14). On the contrary, Fiorenzato et al. reported no cognitive change in MSA-P and MSA-C between baseline and follow-up evaluations.

Because of these controversial aspects concerning CI in MSA, we aimed to retrospectively describe the cognitive profile, its progression, and the relationship with demographic and clinical characteristics of patients with MSA assessed through a comprehensive neuropsychological evaluation and longitudinally followed up at the Department of Biomedical and Neuromotor Sciences (DiBiNeM) at the University of Bologna.

## Materials and Methods

### Patient Selection

We retrospectively selected patients with a final clinical diagnosis of MSA referred to the movement disorders and autonomic disorders centers of our department, between 1991 and 2017, who were evaluated at least once a year during the disease course and who underwent at least one comprehensive neuropsychological evaluation.

Among this group, we also analyzed the data of patients who underwent two neuropsychological evaluations (baseline evaluation = T0 and second evaluation = T1).

Three neurologists specialized in movement disorders independently confirmed MSA diagnosis according to international criteria (1) from data available at the last follow-up evaluation; their consensus and absence of non-supporting features for MSA, excluding the presence of dementia [according to DSM-IV criteria, (15)], were mandatory for inclusion in the study.

### Clinical Features and Instrumental Investigation

The MSA phenotype was defined as cerebellar (MSA-C) or parkinsonian (MSA-P) on the basis of the predominant phenotype at the time of the last follow-up visit. As reported in previous studies (16, 17), we collected the following clinical data from medical records in a standardized fashion by one author and entered them into an *ad hoc* database for statistical analysis: (1) age at disease onset (i.e., age at the time of the first reported motor or autonomic symptom or sign that could be related to MSA); (2) age and cause of death (if applicable); (3) disease duration (i.e., interval from first symptom onset to death or to the last clinical follow-up and neuropsychological evaluation); (4) symptoms at initial presentation; (5) presence of parkinsonian, cerebellar, autonomic, or pyramidal signs at annual neurological evaluations; (6) presence of obstructive sleep apnea syndrome (OSAS), stridor, and/or rapid eye movement (REM) sleep behavior disorder (RBD), confirmed by all-night video polysomnography (VPSG); (7) diagnosis of neurogenic orthostatic hypotension (OH) confirmed by cardiovascular reflex tests; and (8) milestones of disease progression, i.e., frequent falls (at least three falls per year or documentation of frequent or several falls), loss of ambulatory independence, wheelchair dependence, severe dysphagia or percutaneous endoscopic gastrostomy, severe dysarthria, and urinary catheterization. Disease severity was determined based on the number of milestones achieved.

### Neuropsychological Assessment

All patients underwent a comprehensive cognitive assessment composed by an extensive battery of neuropsychological tests, standardized on the Italian population, that examined the main cognitive domains (global cognition, verbal and visual memory, attention, executive and visuospatial function, constructional praxis, and language) (18–30).

Each cognitive domain was evaluated with at least two tests for each function (31). Specifically, global cognition was evaluated with the Mini-Mental State Examination (MMSE) (18, 19) and the Final Result of the Brief Mental Deterioration Battery (FR BMDB) (23); memory with the Rey Auditory Verbal Learning Test (RAVLT)—immediate recall (RAVLT IR) and delayed recall (RAVLT DR) (24); attention with the Barrage test (23) and immediate visual memory (IVM) (24); executive function with the Simple Verbal Analogies Test (SVAT) (23, 32) and Stroop test (25); language with verbal phonemic fluency (VPF) (24) and verbal semantic fluency (FS) tasks (26); and visuospatial and constructive function through simple copy drawing (CD) (24) and pentagon copy (PC) tasks (27). Depression and anxiety were evaluated with the Beck Depression Inventory (BDI) (33) and the State Trait Anxiety Inventory (STAI) (34). We refer to this evaluation as the standard battery.

Because of the retrospective nature of the study, the number of tests used to evaluate each patient was not consistent in all cases. Some of the patients underwent a further evaluation of global cognition with Raven's Progressive Matrices (RPM) (24); memory with Rey's complex figure delayed recall (ReyD) (24) and paired word learning (PWL) (28) tests; working memory with Verbal Span Forward and Backward (SpanVF and SpanVB, respectively) (29, 35) and Corsi's Span (SpanC) (30, 35) tasks; attention and executive function with Trial Making Tests A and B (TMT-A and TMT-B, respectively) (20); and visuospatial functioning with Rey's Figure Copy (ReyC) (21) and Benton Line Orientation (LO) tests (22). These tests constituted the second-level battery.

The comparison of cognitive profiles, frequency of patients, and domains and test on which patients were impaired classified according to the standard battery and the second-level battery did not reveal significant differences in the evaluation (data not shown).

All test results were corrected for age, sex, and education according to Italian standardization.

Normative data were used to define normality and non-normality on each cognitive test.

CI was defined as an abnormal score on at least one test of the standard battery. Mild CI (MCI) was diagnosed according to Litvan et al.'s criteria for MCI in Parkinson's disease level II: objective impairment on at least two tests, either within a single cognitive domain or across different cognitive domains detected by neuropsychological tests with 1.5-SD cut-off, as per the normative Italian data, reported above.

Dementia was defined according to the following criteria: (1) MMSE score (18), corrected for age and education, lower than a value of 23.8, as per Italian standardization (19), and an FR BMDB value lower than 0; and (2) objective impairment in at least four cognitive domains detected by neuropsychological tests with a 1.5-SD cut-off, as per the normative Italian data, reported above.

Worsening or progression of CI was defined if at least a further cognitive domain at T1, with respect to T0 evaluation, was abnormal.

Two neurologists specialized in cognitive disorders independently confirmed the diagnosis of CI, MCI, or dementia. The same neuropsychological evaluation was performed at baseline (T0) and follow-up evaluations (T1).

### Standard Protocol Approval, Registration, and Patient Consent

The ethics committee of Bologna approved the study (AUSL Committee, approval number 17093). All patients provided written informed consent for using personal data for research purposes. The study was performed in accordance with the ethical standards laid down in the 1964 Declaration of Helsinki and its later amendments.

## Statistical Analysis

The normality of the distribution of the continuous parameters was assessed using the skewness–kurtosis test. Continuous variables were compared using Student's test or Mann Whitney *U*-test. Categorical variables were described by their absolute and/or relative frequencies and compared using the chi square test. Because of the retrospective nature of the study, we corrected tests whose performance implied motor ability (i.e., Barrage test, TMT-A, CD, PC, SpanC, ReyC, and RAVLT DR) for the number of milestones achieved. Wilcoxon signed-rank for repeated-measures test was used to compare clinical features and results of cognitive tests at baseline and follow-up evaluations. The Kruskal–Wallis test was used to compare differences among three groups: patients without CI, patients with a stable degree of CI, and patients who progressed from baseline to follow-up evaluations. A correction for multiple comparisons with Bonferroni's method was applied when appropriate. A *p* < 0.05 was considered significant. Statistical analyses were performed using the SPSS (21.0) software package.

## Results

### Clinical, Neuropsychological, and Behavioral Baseline Evaluation

A total of 145 patients with MSA were analyzed. Sixty patients (36 males, mean age at disease onset 57.6 ± 9.3, education 10.3 ± 4.7 years) underwent at least one neuropsychological evaluation and were included in the present study.

Ten of these patients were enrolled in a previous prospective study by our team (12).

Disease duration at neuropsychological evaluation was 5 ± 1 years. These patients did not differ in sex, age at onset, disease duration, or severity from those who did not undergo neuropsychological evaluation (*n* = 85).

The neuropsychological test results of the 60 patients with MSA at baseline evaluation (T0) are presented in Table 1.

**Table 1:** Neuropsychological characteristics of patients.

	**Patients without cognitive impairment** **(*****n*** **=** **23)**		**Patients with cognitive impairment** **(*****n*** **=** **37)**		***p***
	**Mean**	**SD**	**Mean**	**SD**	
MMSE^*^	28.13	1.07	26.22	2.98	0.0001
BMDB^*^	2.24	0.62	1.09	0.93	0.0001
RPM	32.86	1.89	28.71	3.94	0.01
RAVLT DR^*^^§^	44.62	7.94	33.16	7.81	0.0001
RAVLT IR^*^^§^	9.07	2.18	6.87	2.55	0.002
IVM^*^^§^	19.79	2.53	17.77	2.31	0.001
ReyC	33.93	2.49	27.09	9.71	0.22
ReyD	19.09	3.43	13.08	6.05	0.13
PWL	15.34	4.08	8.43	3.27	0.001
SpanVF	6.66	1.17	5.59	1.31	0.09
SpanVB	5.44	0.72	3.69	1.27	0.008
SpanC	5.93	0.82	4.54	1.08	0.004
Barrage^*^^§^	−0.47	0.6	2.08	3.48	0.001
StroopT^*^	18.29	6.92	26.11	9.51	0.11
StroopE^*^	−0.17	0.75	2.43	2.82	0.22
TMT-A	38.15	16.66	59.14	32.99	0.18
VPF^*^	29.6	8.59	24.6	9.01	0.06
VSF^*^^§^	45.78	6.42	42.58	32.2	0.001
SVAT^*^^§^	18.22	1.39	15.2	3.79	0.001
TMT-B	56.4	42.60	114.30	59.93	0.009
TMT-B-A	19.15	30.16	53.34	41.73	0.03
CD^*^	11	1	7.6	4.39	0.09
LO	28.00	2.07	23.33	5.07	0.02
BDI	12.24	6.96	18.05	11.23	0.06
STAI	41	11.38	47	19	0.23

MMSE, Mini-Mental State Examination; BMDB, Brief Mental Deterioration Battery; RPM, Raven's Progressive Matrices; RAVLT IR, Rey Auditory Verbal Learning Test, immediate recall; RAVLT DR, Rey Auditory Verbal Learning Test, delayed recall; IVM, Immediate visual memory; ReyC, Rey's Figure Copy; ReyD, Rey's complex figure delayed recall; PWL, Paired word learning; SpanVF, Verbal Span Forward; SpanVB, Verbal Span Backward; SpanC, Corsi's Span; Barrage, Barrage test; TMT-A, Trial Making Test A; TMT-B, Trial Making Test B; TMT B-A, Trial Making Test B-A; Stroop test T, Stroop Test, Time; Stroop test E, Stroop Test, Error; SVAT, Simple Verbal Analogies Test; VPF, Verbal Phonemic Fluency; VSF, Verbal Semantic Fluency; CD, Simple Copy Drawing; LO, Benton Line Orientation; BDI, Beck Depression Inventory; STAI, State Trait Anxiety Inventory.

* The test included in the standard battery of cognitive evaluation used in our center.

§ *Significantly different results (p < 0.05)*.

**Figure 1:**
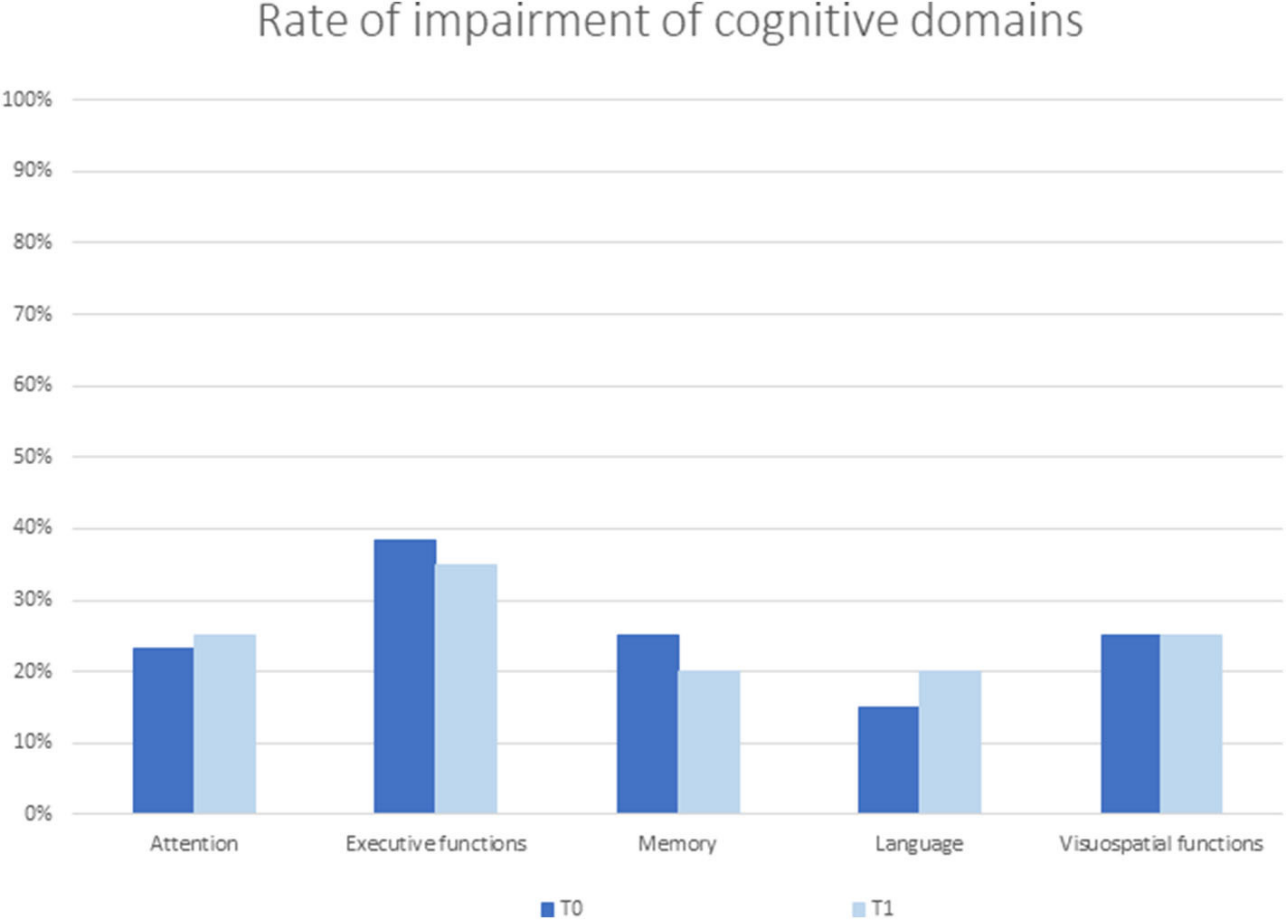
Rate of impairment of cognitive domains at baseline (TO) and follow-up (T1) evaluation.

Thirty-seven out of 60 patients (62%) were cognitively impaired. At neuropsychological evaluation, patients with and without CI did not significantly differ in age (61.73 ± 14.29 and 60.26 ± 7.18 years, respectively; *p* = 0.20) and sex (M/F: 25/12 and 11/12, respectively; *p* = 0.18), while education was significantly lower in patients with CI (8.9 ± 4.4 vs. 12.5 ± 4.3 years in patients without CI; *p* < 0.003). Of the 60 patients, executive function was impaired in 23 (38%), verbal memory and visuospatial functions in 15 (25%), attention in 14 (23%), and language in 9 (15%) patients (Figure 1).

Comparing the results on each task, we found that patients with CI had significantly worse performance compared to those without CI mainly on tests of attention and executive function (Barrage total score, *p* < 0.0002, and SVAT, *p* < 0.001), followed by performance on tests of memory (RAVLT IR, *p* < 0.0001; RAVLT DR, *p* < 0.002; and IVM, *p* < 0.001) and semantic fluency (*p* < 0.001). No differences were detected on the affective tasks (depression, *p* = 0.06, and anxiety, *p* = 0.23). Thirteen out of 37 (35%) patients were impaired in one cognitive domain and 24 (65%) in more than one cognitive domain. Eighteen patients met the criteria for MCI. They were all impaired in attention and executive functioning; nine patients were also impaired in memory and praxis.

Patients with MCI had significantly lower education (mean ± SD: 8.87 ± 4.79), higher age at onset (mean ± SD: 59.04 ± 9.55), and worse cognitive performance on all evaluation measures except on number of errors on the Stroop test (mean ± SD: 0.18 ± 0.71) and CD (mean ± SD: 7.25 ± 4.99) compared to patients without CI. No other clinical or demographic differences were observed.

Six out of 24 (25%) patients were classified as having dementia. Patients with dementia (4 M/2 F; age at disease onset 62.5 ± 3.8 years; age at neuropsychological evaluation 68.5 ± 3.3 years; disease duration 54.9 ± 12.4 months; MSA-P = 2, MSA-C = 4) had higher age at disease onset and at neuropsychological evaluation and lower education and disease duration at neuropsychological evaluation than the other group of patients.

No differences in the cognitive profile were detected between patients who underwent the standard (*n* = 42) and the second-level neuropsychological evaluations (*n* = 18).

Clinical variables (i.e., clinical phenotype at onset, disease duration, and severity) and autonomic or sleep disturbances (OH, RBD, OSAS, and stridor) were not different between patients with and without CI (Table 2).

**Table 2:** Clinical characteristics of patients.

	**Patients without cognitive impairment** **(*n* = 23)**	**Patients with cognitive impairment** **(***n* = 37^*^)	***p***
Age at onset (years)	55.91 ± 7.92	58.54 ± 10.02	0.62
Disease duration (months)	56.54 ± 38.32	62.01 ± 36.03	0.73
Deceased patients	13	16	0.52
Phenotype MSA P/C	10/13	19/18	0.51
MSA-P Possible	3	2	
MSA-P Probable	7	17	
MSA-C Possible	1	4	
MSA-C Probable	12	14	
Parkinsonism	16	27	0.22
Cerebellar signs	17	28	0.96
Pyramidal signs	15	25	0.74
OH	17	27	0.26
Urinary disturbances	18	30	0.65
RBD	12^#^	23	0.26
OSAS	5	8	0.34
Stridor	6	9	0.58
Dysarthria	8	12	0.67
Disease severity			0.08
0	7	10	
1	7	6	
2	3	12	
3	6	2	
4	0	3	
5	0	2	

MSA, multiple system atrophy; P, parkinsonian type; C, cerebellar type; OH, orthostatic hypotension; RBD, REM sleep behavior disorder; OSAS, obstructive sleep apnea syndrome. 0–5: number of milestones of progression.

* The number includes patients with dementia (n = 6).

# *Two patients showed RSWA (REM sleep without atonia) at video polysomnography*.

In detail, mean blood pressure values continuously recorded during supine position and head up tilt test were not significantly different between cognitively normal and cognitively impaired patients.

### Neuropsychological and Behavioral Follow-Up Evaluation

Twenty patients underwent a follow-up evaluation (T1) after a mean of 16.6 ± 9.3 months from the first evaluation (T0) (age at T1 = 60.9 ± 9.5 years; disease duration = 82 ± 46.2 months). These patients did not differ in demographic, clinical, and neuropsychological variables from those who did not undergo neuropsychological evaluation at follow-up (*n* = 40).

**Figure 2:**
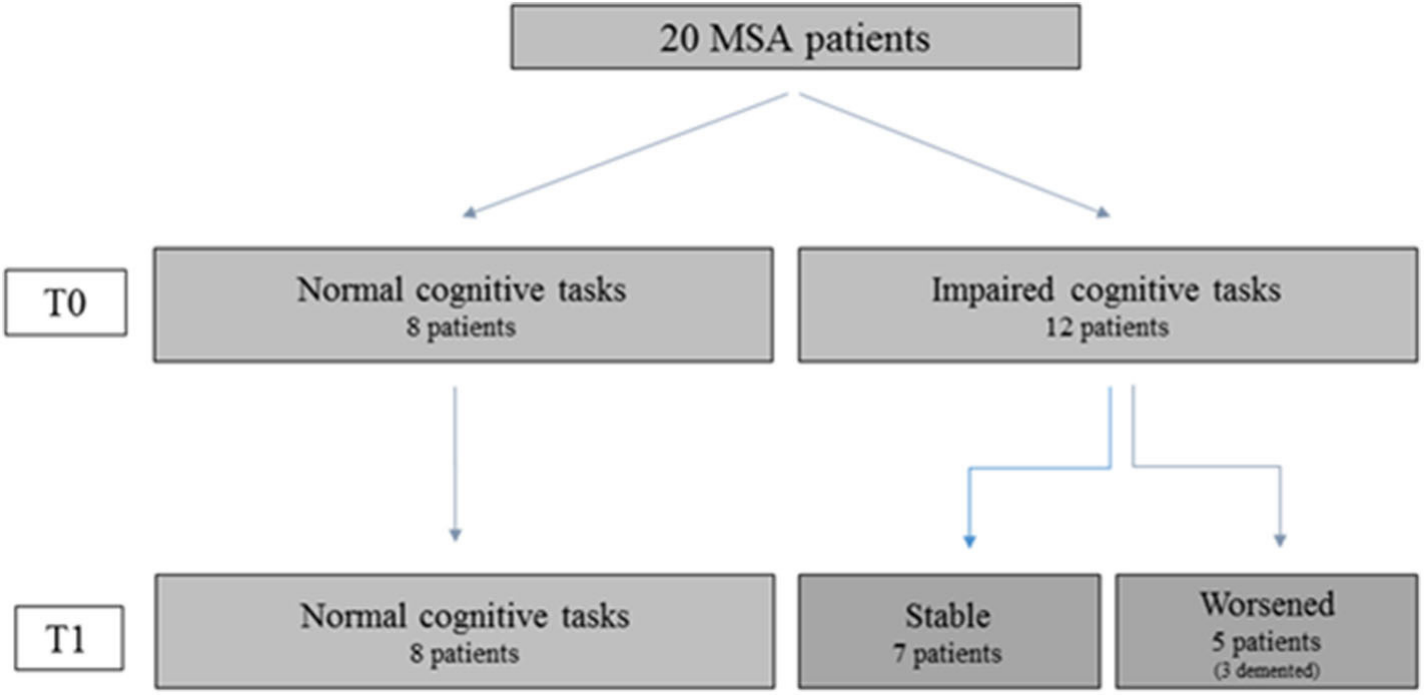
Cognitive performances of patients who performed both baseline (TO) and follow-up (T1) assessment. The figure shows that in MSA patients' cognitive impairment can be stable or worsen along the disease course. Hence the figure suggests that in order to characterize the cognitive profile and evaluate its possible evolution toward dementia both the cross-sectional observation (TO) and follow-up assessment (T1) are necessary.

Single-subject analysis showed that eight patients remained without CI both at T0 and at T1; seven had a stable CI, while five progressed at T1 (Figure 2). The three groups had similar demographic and clinical characteristics. In contrast, at the baseline evaluation, patients who progressed performed significantly worse on global functioning (FR BMDB, *p* < 0.001, and MMSE, *p* < 0.002); verbal memory, both short (*p* < 0.001) and long term (*p* < 0.01); semantic fluency (*p* < 0.002); and visuospatial functioning (*p* < 0.002) compared to cognitively stable patients (both with and without CI). These differences remained at the follow-up evaluation (Table 3). At T1, the main domains of worsening were attention and executive functions and, to a lesser extent, memory and visuospatial functions. Three patients developed dementia. Affective ratings did not differ between the three groups and between baseline and follow-up observations.

**Table 3:** Neuropsychological evaluation at follow-up.

	**Patients without cognitive impairment** **(*****n*** **=** **8)**		**Cognitively stable patients** **(*****n*** **=** **7)**		**Patients with cognitive worsening** **(*****n*** **=** **5)**	
	**Mean**	**SD**	**Mean**	**SD**	**Mean**	**SD**
MMSE^*^^§^	29.00	1.54	27.34	2.12	24.76	3.39
BBDM^*^^§^	2.77	0.58	1.92	0.82	0.36	1.37
RPM	33.76	1.54	30.07	6.54	22.05	1.06
RAVLT IR^*^^§^	48.59	9.65	39.89	7.12	29.83	9.80
RAVLT DR^*^^§^	10.56	2.10	8.54	4.13	4.91	1.45
IVM^*^	20.54	1.98	19.16	1.43	18.34	3.72
ReyC	34.16	2.42	27.09	8.94	4.28	3.15
ReyD	19.35	2.41	15.22	4.16	3.75	–
PWL	15.09	2.67	10.74	3.10	6.58	3.49
SpanVF	6.33	1.06	7.75	–	6.38	1.24
SpanVB	5.22	1.03	6	–	4.48	2.11
SpanC	6.04	0.28	5.54	1.25	3.88	1.59
Barrage^*^	−0.46	0.61	0.57	0.97	2.51	2.31
StroopT^*^	14.72	6.98	17.34	5.28	47.28	35.11
TMT-A	46.50	17.10	49.50	0.87	98.17	83.29
VPF^*^	31.31	9.58	25.07	5.31	22.17	10.25
VSF^*^^§^	47.00	5.39	37.57	3.82	27.40	6.80
SVAT^*^	19.00	1.07	17.91	1.76	13.60	4.05
TMT-B	62.17	36.55	88.50	16.04	187.67	149.15
TMT B-A	15.67	22.18	37.17	20.87	88.67	79.35
CD^*^^§^	11.36	0.77	9.65	1.56	6.61	2.79
LO	27.50	1.87	24.00	6.56	14.00	2.83
BDI	9.87	4.99	19.83	12.31	7.33	7.02
STAI	49.43	11.35	54.60	10.69	53.50	7.77

MMSE, Mini-Mental State Examination; BMDB, Brief Mental Deterioration Battery; RPM, Raven's Progressive Matrices; RAVLT IR, Rey Auditory Verbal Learning Test, immediate recall; RAVLT DR, Rey Auditory Verbal Learning Test, delayed recall; IVM, Immediate visual memory; ReyC, Rey's Figure Copy; ReyD, Rey's complex figure delayed recall; PWL, Paired word's learning; SpanVF, Verbal Span Forward; SpanVB, Verbal Span Backward; SpanC, Corsi's Span; Barrage, Barrage test; TMT-A, Trial Making Test A; TMT-B, Trial Making Test B; TMT B-A, Trial Making Test B-A; Stroop test T, Stroop Test, Time; Stroop test E, Stroop Test, Error; SVAT, Simple Verbal Analogies Test; VPF, Verbal Phonemic Fluency; VSF, Verbal Semantic Fluency; CD, Simple Copy Drawing; LO, Benton Line Orientation; BDI, Beck Depression Inventory; STAI, State Trait Anxiety Inventory.

* The test included in the standard battery of cognitive evaluation used in our center.

§ *Significantly different results (p < 0.05). – SD is missing as only one patient performed the test*.

## Discussion

Our study describes the cognitive profile and its evolution in a large cohort of patients with MSA, as assessed through a comprehensive neuropsychological battery and observed at the long disease duration and for the long follow-up period. We reported the frequency and profile of CI according to the clinical criteria for dementia and CI in our cohort of patients with MSA. Furthermore, patients with MSA included in this study were frequently evaluated (at least once a year) during the course of the disease with a comprehensive neurological examination. More than one third (38%) of our patients were without CI at baseline neuropsychological evaluation, while among 37 patients with CI (62%), 13 patients (35%) showed abnormal results in one cognitive domain and 24 (65%) in more than one cognitive domain.

A few previous studies (11, 36), including a more recent retrospective multicenter study (37), showed a higher prevalence of patients impaired in a single cognitive domain and a decreasing frequency of impairment when the number of domains increased. Studies of pathologically proven patients with MSA reported a mild to moderate CI, assessed through bedside evaluation in 22 and 2% of patients, respectively (38), and a frequency of impairment from 25 to 39% according to a physician's observation or patient or caregiver complaints (38, 39). This variable frequency could be explained by the different criteria and tests used, as suggested in a recent systematic review (5).

Regarding the impairment of specific functions, in our cohort, three quarters (76%) of the patients were selectively impaired in attention or executive functioning. In the remaining patients, cognitive deficit was associated with abnormal memory and/or visuospatial functioning. These findings are consistent with several previous studies (11, 12, 36, 40), including the only study that reported the frequency of impairment in each specific cognitive domain in pathologically proven patients with MSA (38). Other studies evaluated only one to three domains (6, 41–44) or reported the overall performance on tests or group scores without referring to the pattern of impairment of cognitive functioning (45–48). Furthermore, some studies reported that these scores were still within normal ranges compared to the scores of control groups (8, 43).

Consistent with existing evidence (3, 37), the worst cognitive profile, compatible with dementia, was observed in 16% (6/37) of this cohort. In pathologically proven patients with MSA, dementia, diagnosed based on bedside evaluation, was observed only in one patient (0.5%) (49).

Our study also compared the demographic and clinical variables, including both motor and non-motor domains, in patients with and without CI. Consistent with a previous study of patients with MSA with confirmed diagnosis (36), we documented that education was significantly lower in patients with CI and that patients with dementia were older at disease onset compared to the other patients. In contrast, other smaller studies did not document any significant differences in these variables (3, 38, 44). Other demographic and clinical variables were similar among the groups.

Consistent with three previous studies (10, 36, 37), we found no differences in cognitive profiles between MSA-P and MSA-C patients. In contrast, Balas et al. found different cognitive performances in patients with MSA-P and MSA-C compared to controls (43). Similarly, Kawai et al. reported that patients with MSA-P showed severe impairment in visuospatial and constructional functions, verbal fluency, and executive function compared to patients with MSA-C and controls (44). Further, Chang et al. reported that patients with MSA-C presented with a more pronounced executive and verbal memory decline compared with patients with MSA-P (47). These discrepancies may be related to differences in study design, sample size, evaluation methods, and diagnostic criteria of CI; confounding factors that can modify the cognitive performance, in this disease mainly OH (48, 50); or treatment effects. In our study, we did not evaluate the potential effect of the modification of cerebral autoregulation on cognitive function (51).

Considering non-motor domains, only Brown et al. reported that cardiovascular dysautonomia is an independent predictor of CI in patients with MSA (36). However, genitourinary dysautonomia did not predict CI. In contrast, our patients, with and without CI, did not show differences in autonomic dysfunction or sleep disturbances, associated with the presence of motor and respiratory disorders during sleep.

Finally, we evaluated the progression of CI in patients with MSA. At the follow-up evaluation, a quarter of the patients had a clinically meaningful decline in cognitive performance. At baseline evaluation, these patients performed worse in global functioning; verbal memory, both short and long term; semantic fluency; and visuospatial functioning compared to cognitively stable patients. This suggests that patients who progress toward dementia at baseline have a different cognitive profile compared to cognitively stable patients. Furthermore, none of the patients with preserved cognitive function at baseline developed cognitive deficits during the follow-up period. To date, only a few studies have evaluated the evolution of cognitive functioning in patients with MSA and found a worsening of attention and executive functioning.

## Conclusions

In conclusion, our findings indicate that the cognitive profile of patients with MSA can be characterized by normal cognitive functioning even in the sixth year of the disease or by a selective and stable impairment of attention and executive functions that could be related to fronto-striatal-cerebellar dysfunction. This finding supports the idea that a specific pattern of CI is an integral part of MSA phenotype, which probably reflects the direct consequence of both cortical and subcortical atrophies and their associated cortical pathophysiological change (3, 13, 40, 44, 52).

A less common MSA cognitive phenotype could be characterized by a progressive attention–executive dysfunction associated with memory and visuospatial impairment that evolves over time into dementia. This phenotype can be related to a multifactorial neuropathological process with involvement of limbic regions in addition to the other classical regions due to Lewy body spectrum pathology or rarely to advanced Alzheimer's disease neuropathology or hippocampal sclerosis (39, 53, 54).

Our results demonstrate that the detection of CI in patients with suspected MSA does not exclude the diagnosis but suggests the need for a comprehensive neuropsychological evaluation aimed at characterizing the deficit (55). Furthermore, in the advanced phases of the disease, cognitive abnormalities may occur with the proper features of dementia and thus must be readily recognized and framed.

In addition, cognitive deficits are not related to motor disturbances and autonomic dysfunction.

The main strengths of our study are the large size of the sample; the monocentric evaluation of patients; the annual update of information at every follow-up visit, which allowed the evaluation of the evolution of CI in patients with MSA; and the comprehensive clinical and diagnostic examination. Our results suggest a need for a specific neuropsychological evaluation that takes into consideration the mnestic–linguistic–praxic functions in addition to the commonly evaluated attentive and executive functions. The assessment should be especially suitable for advanced stages of the disease, as dysarthria and akinesia may interfere with performance on neuropsychological tests that involve a motor or timed task (55).

The main limitations of the study are its retrospective nature, lack of assessment of activities of daily living, and the lack of pathophysiological data (i.e., neuroimaging, neurophysiological, and neuropathological data).

## Data Availability Statement

The datasets generated for this study are available on request to the corresponding author.

## Ethics Statement

The studies involving human participants were reviewed and approved by AUSL Committee, number of approval 17093. The patients/participants provided their written informed consent to participate in this study.

## Author Contributions

LS: conceptualization, formal analysis, data maturation, writing original draft, review, and editing. GC-B and IC: conceptualization, formal analysis, writing original draft, review, and editing. GG and MS: conceptualization, review, and editing. FP: conceptualization. RP and FO: conceptualization and data curation. PC: supervision, funding acquisition, conceptualization, review, and editing. All authors contributed to the article and approved the submitted version.

## Conflict of Interest

FP received honoraria for speaking engagements or consulting activities from Sanofi, Bial, Fidia, and Vanda Pharmaceutical. PC received honoraria for speaking engagements or consulting activities from Allergan Italia, AbbVie srl, Chiesi Farmaceutici, Eli Lilly, Novartis, Teva, UCB Pharma S.p.A, and Zambon. The remaining authors declare that the research was conducted in the absence of any commercial or financial relationships that could be construed as a potential conflict of interest.
